# Comparative Study of Immunogenic Properties of Purified Capsular Polysaccharides from *Streptococcus suis* Serotypes 3, 7, 8, and 9: the Serotype 3 Polysaccharide Induces an Opsonizing IgG Response

**DOI:** 10.1128/IAI.00377-20

**Published:** 2020-09-18

**Authors:** Guillaume Goyette-Desjardins, Jean-Philippe Auger, Dominic Dolbec, Evgeny Vinogradov, Masatoshi Okura, Daisuke Takamatsu, Marie-Rose Van Calsteren, Marcelo Gottschalk, Mariela Segura

**Affiliations:** a Swine and Poultry Infectious Diseases Research Centre, Faculty of Veterinary Medicine, University of Montreal, Saint-Hyacinthe, Quebec, Canada; b Research Group on Infectious Diseases in Production Animals, Faculty of Veterinary Medicine, University of Montreal, Saint-Hyacinthe, Quebec, Canada; c Canadian Glycomics Network (GlycoNet), University of Alberta, Edmonton, Alberta, Canada; d National Research Council, Ottawa, Ontario, Canada; e Division of Bacterial and Parasitic Disease, National Institute of Animal Health, National Agriculture and Food Research Organization, Tsukuba, Ibaraki, Japan; f The United Graduate School of Veterinary Sciences, Gifu University, Gifu, Gifu, Japan; g Saint-Hyacinthe Research and Development Centre, Agriculture and Agri-Food Canada, Saint-Hyacinthe, Quebec, Canada; Washington State University

**Keywords:** *Streptococcus suis*, capsular polysaccharide, immunogenicity, serotype 3

## Abstract

Streptococcus suis is an encapsulated bacterium and one of the most important swine pathogens and a zoonotic agent for which no effective vaccine exists. Bacterial capsular polysaccharides (CPSs) are poorly immunogenic, but anti-CPS antibodies are essential to the host defense against encapsulated bacteria. In addition to the previously known serotypes 2 and 14, which are nonimmunogenic, we have recently purified and described the CPS structures for serotypes 1, 1/2, 3, 7, 8, and 9. Here, we aimed to elucidate how these new structurally diverse CPSs interact with the immune system to generate anti-CPS antibody responses. CPS-stimulated dendritic cells produced significant levels of C–C motif chemokine ligand 3 (CCL3), partially via Toll-like receptor 2 (TLR2)- and myeloid differentiation factor 88-dependent pathways, and CCL2, via TLR-independent mechanisms. Mice immunized with purified serotype 3 CPS adjuvanted with TiterMax Gold produced an opsonizing IgG response, whereas other CPSs or adjuvants were negative. Mice hyperimmunized with heat-killed S. suis serotypes 3 and 9 both produced anti-CPS type 1 IgGs, whereas serotypes 7 and 8 remained negative. Also, mice infected with sublethal doses of S. suis serotype 3 produced primary anti-CPS IgM and IgG responses, of which only IgM were boosted after a secondary infection. In contrast, mice sublethally infected with S. suis serotype 9 produced weak anti-CPS IgM and IgG responses following a secondary infection. This study provides important information on the divergent evolution of CPS serotypes with highly different structural and/or biochemical properties within S. suis and their interaction with the immune system.

## Abstract (precis)

Streptococcus suis is an encapsulated bacterium and one of the most important swine pathogens and a zoonotic agent for which no effective vaccine exists. Bacterial capsular polysaccharides (CPSs) are poorly immunogenic, but anti-CPS antibodies are essential to the host defense against encapsulated bacteria. In addition to the previously known serotypes 2 and 14, which are nonimmunogenic, we have recently purified and described the CPS structures for serotypes 1, 1/2, 3, 7, 8, and 9.

## INTRODUCTION

Streptococcus suis is an encapsulated Gram-positive bacterium and is considered to be one of the most important causes of bacterial infection and death in piglets postweaning. This infection is responsible for important economic losses to the pig industry. Moreover, infections by this pathogen compromise animal welfare. Striking manifestations of the disease in pigs are septicemia and meningitis, but other clinical manifestations can also be present as endocarditis and arthritis. S. suis is mainly localized in the upper respiratory tracts of pigs, more particularly the tonsils and nasal cavities, and it may be acquired vertically from the sows or horizontally through piglet-to-piglet transmission. While close to 100% of pig farms globally have carrier animals, only a limited number of animals develop clinical disease in the presence of prophylactic medication (1). However, the recent global trend of enforced reduction in antimicrobial use in livestock production has resulted in an alarming increase of S. suis clinical disease in swine herds. S. suis is also an emerging zoonotic pathogen, leading to meningitis, septic shock, and other less common clinical manifestations usually associated with generalized septicemia. This zoonosis is of particular worldwide incidence in people working in close contact with infected pigs and/or pork-derived products during their professional activities (2). Most importantly, even the general population is at risk in some Asian countries, where multiple outbreaks of infection have been reported after ingestion of contaminated raw pork products (2, 3). Indeed, in countries such as Vietnam and Thailand, S. suis is considered to be among the most frequent causes of bacterial meningitis in human adults (4). Sadly, to this day, there is no effective commercial vaccine to fight this infection in either swine or humans (4).

One of the most important virulence factor of S. suis is its cell-associated capsular polysaccharide (CPS), a feature in agreement with other encapsulated pathogens such as Streptococcus pneumoniae and group B *Streptococcus* (GBS) (5, 6). As a matter of fact, the initially described 35 serotypes of S. suis were established according to CPS antigenicity. Worldwide, the top 10 predominant serotypes in S. suis isolates reported between 2002 and 2013 from clinical cases in swine are, in decreasing order, serotypes 2, 9, 3, 1/2, 8, 7, 4, 22, 5, and 1 (2). In humans, while the vast majority of infections are caused by serotype 2 and, to a smaller extent, by serotype 14, isolated cases triggered by serotypes 1 and 9 strains have also been reported (2, 7, 8).

It is well known that the thick layer of surface-associated CPS protects S. suis against the immune response, notably by conferring resistance to phagocytosis (6, 9). Thus, as with other encapsulated pathogens such as GBS, S. pneumoniae, Neisseria meningitidis, and Haemophilus influenzae, antibodies targeting the CPS are particularly opsonizing and mediate protection (10–12).

Dendritic cells (DCs), the most potent antigen-presenting cells (APCs), express varied pattern recognition receptors (PRRs) that allow them to sense the presence of numerous pathogens via the recognition of pathogen-associated molecular patterns (PAMPs). Among these PRRs, Toll-like receptors (TLRs) contribute to the activation of the innate immune response and help influencing the development of the adaptive immunity (13). Adaptive immune responses are strongly influenced by the interactions between pathogens and DCs, notably by the release of cytokines (14). Nevertheless, due to their carbohydrate composition, bacterial CPSs are commonly considered poorly immunogenic due to their inability to engage T cell help for B cell optimal activation, classifying them as T cell-independent (TI) antigens (15). While numerous *in vitro* studies have established the ability of bacterial CPSs to interact with APCs, ensuing cytokine and chemokine production (16–20), others have linked this production with the presence of bacterial contaminants, such as TLR ligands, in purified CPS preparations (15, 21, 22). Nevertheless, TLR2 and the adaptor molecule myeloid differentiation factor 88 (MyD88), have been assumed to be implicated at the interface of bacterial CPSs and APCs, leading to effective antibody responses (15, 17, 20, 21).

**FIG 1:**
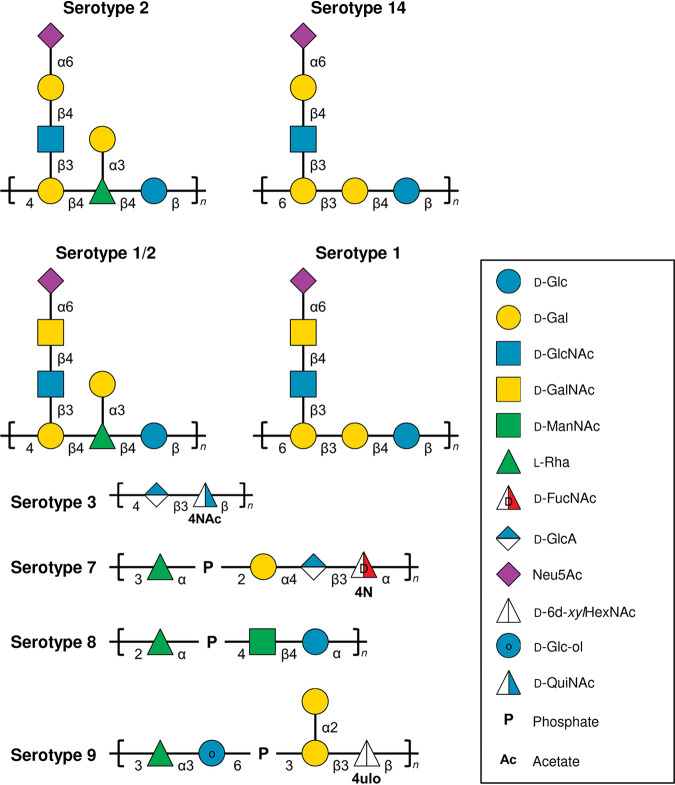
Repeating unit structures of S. suis capsular polysaccharides that were included in this study: serotypes 2 (23), 14 (24), 1/2 and 1 (28), 3 (30), 7 and 8 (31), and 9 (29). Monosaccharide symbols follow the Symbol Nomenclature for Glycans (SNFG) system (79). Abbreviation: 2-acetamido-2,6-dideoxy-β-d-*xylo*-hexopyranose (d-6d*xyl*HexNAc).

The repeating unit structure for S. suis serotype 2 CPS (CPS 2) has been reported in 2010, followed by that for serotype 14 in 2013 (Fig. 1) (23, 24). Working with those two serotypes, Calzas et al. first demonstrated that stimulation of DCs with purified CPSs induced no cytokine production but led to an important production of C–C motif chemokine ligand 2 (CCL2) and CCL3 (25). Second, they demonstrated a very low or undetectable primary anti-CPS IgM response with only a moderate boost in IgM and no isotype switching as features of the antibody response following mouse infections (26, 27). Third, they have been unable to measure anti-CPS antibody responses *in vivo* or anti-CPS antibody-secreting cells *ex vivo* following mouse immunization with purified S. suis serotype 2 or 14 CPSs (27).

Since then, our team, which is the only one to report and expand the known CPS structures of S. suis, has published complete structural determination for the CPSs of serotypes 1 and 1/2 (28), serotype 9 (29), serotypes 3 and 18 (30), and serotypes 7 and 8 (31). Serotype 2, 14, 1, and 1/2 CPSs are structurally related, having similar compositions. They all contain terminal α-Neu5Ac linked to O-6 of β-Gal or β-GalNAc. Serotypes 2 and 14 both possess a β-galactose (Gal) in their side chains that is replaced by *N*-acetylgalactosamine (GalNAc) in serotypes 1 and 1/2 (Fig. 1) (28). This substitution has been linked to a single amino acid polymorphism in the glycosyltransferase CpsK that defines the enzyme substrate predilection for Gal or GalNAc and therefore determines CPS composition, structure, and strain serotype (32, 33). Thus, our first hypothesis is that the CPSs of serotypes 1 and 1/2 are as nonimmunogenic as those of serotypes 2 and 14 due to their structural similarity. CPSs of serotypes 3, 7, 8, and 9, on the other hand, are structurally well diversified among themselves and from those of the previous four serotypes: they do not possess sialic acid, some contain phosphates in their backbone, and others contain unusual sugars such as di-*N*-acetylbacillosamine (QuiNAc4NAc), 4-amino-4-deoxy-*N*-acetylfucosamine (Fuc2NAc4N), glucitol, and a 4-keto sugar (6d*xylo*HexNAc-4-ulose) (Fig. 1). In addition, the CPS of S. suis serotype 8 has been shown to be identical to that of S. pneumoniae serotype 19F (Fig. 1) (31, 34–36). Pneumococcal serotype 19F CPS is considered to be immunogenic, since it is included in the 23-valent pneumococcal polysaccharide vaccine (37). Accordingly, our second hypothesis is that, due to their structural differences, the CPSs of serotypes 3, 7, 8, and 9 have the potential to differently modulate DC activation and the subsequent humoral immune response. Therefore, using purified CPSs, we aimed to study their ability to stimulate APCs *in vitro* and to perform *in vivo* immunogenicity studies with the newly described serotypes 1, 1/2, 3, 7, 8, and 9 of S. suis.

## RESULTS

### *S. suis* CPS purification and quality controls.

In order to perform immunogenicity testing of purified CPSs of S. suis serotypes 2, 1, 1/2, 3, 7, 8, and 9, reference and field strains (Table 1) were used for the CPS production. Quality controls of polysaccharides were performed to ensure the absence of immunogenic contaminants that may bias our study (Table 2). Results revealed that <1% (wt/wt) of nucleic acids and proteins were detected in the purified CPS samples, with the only exception being CPS 9, which showed a striking protein content of 6.7% (wt/wt). One possible explanation for that unusual result is that the modified Lowry assay is subject to interference by substances with reducing potential, such as the unique 4-keto sugar of CPS 9 (2-acetamido-2,6-dideoxy-β-d-*xylo*-hexopyranos-4-ulose). To test this hypothesis, a NanoOrange assay was performed on selected samples, since this assay uses a hydrophobic fluorescent dye and possesses a sensitivity similar to the modified Lowry assay (Table 2). Using the NanoOrange assay, no proteins were found above the detection limit of 0.4% (wt/wt), confirming that CPS 9 is indeed free from protein and also that the result obtained with the modified Lowry assay is due to an interference by its 4-keto sugar.

**TABLE 1:** Bacterial strains of Streptococcus suis used in this study

Strain	Serotype	General characteristics	Used for	Source or reference(s)
S735 (ATCC 43765)	2	Reference strain for serotype 2 CPS production.	CPS purification	23, 80
		Strain isolated from a clinical case of swine infection in Denmark	Mouse hyperimmunization	
P1/7 (ATCC BAA-853)	2	Well-encapsulated serotype 2 strain isolated from a clinical case of swine infection in the United Kingdom (wild-type strain)	Mouse sublethal infections	81
SS2to3	3	CPS-switched mutant derived from P1/7 strain; the *cps-2* locus was genetically excised and replaced with the *cps-3* locus	Mouse sublethal infections	This study
1178027	1	Field strain for serotype 1 CPS production.	CPS purification	28
		Strain isolated from a clinical case of swine infection in Canada		
2651	1/2	Reference strain for serotype 1/2 CPS production. Strain isolated from a clinical case of swine infection in Denmark	CPS purification	28, 80
4961	3	Reference strain for serotype 3 CPS production.	CPS purification	30, 80
		Strain isolated from a clinical case of swine infection in Denmark	Mouse hyperimmunization	
			Opsonophagocytosis assay	
			Mouse sublethal infections	
1750775	7	Field strain for serotype 7 CPS production.	CPS purification	31
		Strain isolated from a clinical case of swine infection in Canada	Mouse hyperimmunization	
			Coating of ELISA plates with S. suis	
1719887	8	Field strain for serotype 8 CPS production.	CPS purification	31
		Strain isolated from a clinical case of swine infection in Canada	Mouse hyperimmunization	
			Coating of ELISA plates with S. suis	
1273590	9	Field strain for serotype 9 CPS production.	CPS purification	29
		Strain isolated from a clinical case of swine infection in Canada	Mouse hyperimmunization	
			Coating of ELISA plates with S. suis	
1135776	9	Well-encapsulated serotype 9 field strain isolated from a clinical case of swine infection in Canada	Mouse sublethal infections	82

**TABLE 2:** Quality control tests of purified S. suis and S. pneumoniae CPSs

CPS	% (wt/wt)		
	Nucleic acids^*a*^	Proteins	
		Modified Lowry assay^*b*^	NanoOrange assay^*c*^
S. suis serotype 2	0.4	<0.5	<0.4
S. suis serotype 1	0.4	0.6	ND
S. suis serotype 1/2	0.2	<0.5	ND
S. suis serotype 3 native	0.4	0.5	<0.4
S. suis serotype 3 phenol-extracted	0.2	<0.5	<0.4
S. suis serotype 7	0.2	<0.5	ND
S. suis serotype 8	0.2	<0.5	ND
S. suis serotype 9	0.5	6.7	<0.4
S. pneumoniae serotype 19F	0.2	<0.5	ND

a Determined by spectrophotometry at 230 and 260 nm.

b Determined by using a modified Lowry protein assay kit. Values that were below the limit of detection are shown as <0.5% (wt/wt).

c Determined by using a NanoOrange protein quantitation kit. Values that were below the limit of detection are shown as <0.4% (wt/wt). ND, not determined.

### *S. suis* CPSs induce the release of chemokines by DCs.

**FIG 2:**
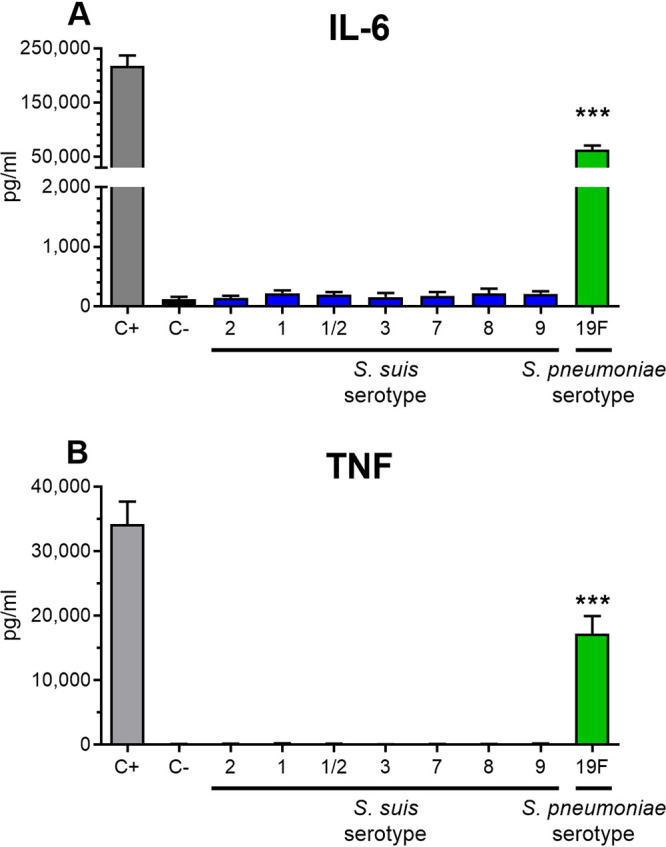
Proinflammatory cytokine production by DCs in response to stimulation by S. suis or S. pneumoniae CPSs for 24 h. Native CPSs of S. suis serotypes 2, 1, 1/2, 3, 7, 8, and 9 or of S. pneumoniae serotype 19F (each at 200 μg/ml) were incubated with DCs (10^6^ cells/ml). After 24 h, supernatants were collected, and the IL-6 (A) and TNF (B) levels were quantified by ELISA. Cells stimulated with medium alone and with LPS (1 μg/ml) served as negative (C–) and positive (C+) controls, respectively. The data are expressed as means ± the SEM for at least three experiments. Statistically significant differences versus medium alone (C–) are indicated (***, *P ≤ *0.001).

To study the ability of purified CPSs of S. suis to stimulate APCs *in vitro*, mouse bone marrow-derived DCs were chosen. Here, after a 24-h stimulation of DCs with 200 μg/ml for each S. suis CPS, the levels of two proinflammatory cytokines in the supernatant, namely, interleukin-6 (IL-6) and tumor necrosis factor (TNF), were measured. No significant difference in cytokine production was observed between DCs incubated with the different CPSs and those incubated with medium alone (*P* > 0.05) (Fig. 2). For reference, purified S. pneumoniae serotype 19F CPS preparation for vaccine production was purchased and used to stimulate DCs in the same manner. As shown in Fig. 2, pneumococcal CPS 19F induced strong production of both IL-6 and TNF.

**FIG 3:**
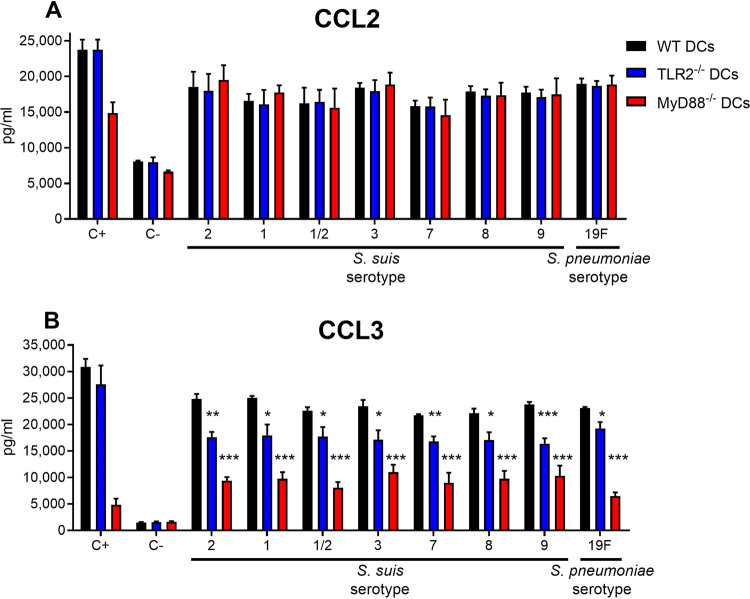
Role of TLR2 and of MyD88 in chemokine production by DCs in response to stimulation by S. suis or S. pneumoniae CPSs for 24 h. Native CPSs of S. suis serotypes 2, 1, 1/2, 3, 7, 8, and 9 or of S. pneumoniae serotype 19F (each at 200 μg/ml) were incubated with either wild-type (WT; black bars), TLR2^−/−^ (blue bars), or MyD88^−/−^ (red bars) DCs (10^6^ cells/ml). After 24 h, supernatants were collected, and CCL2 (A) and CCL3 (B) levels were quantified by ELISA. Cells stimulated with medium alone and with LPS (1 μg/ml) served as negative (C–) and positive (C+) controls, respectively. The data are expressed as means ± the SEM for at least three experiments. Statistically significant differences *versus* the WT are indicated (*, *P ≤ *0.05; **, *P ≤ *0.01; ***, *P ≤ *0.001).

In contrast to these proinflammatory cytokines, all S. suis and S. pneumoniae CPSs at 200 μg/ml induced significant release of the chemokines CCL2 and CCL3 after incubation with DCs (Fig. 3). Thus, we also investigated the involvement of PRRs in chemokine release by CPS-stimulated DCs by comparing wild-type (WT) DCs with either TLR2^−/−^ or MyD88^−/−^ DCs. The results showed no significant difference in CCL2 production (Fig. 3A). This indicates that CCL2 production by DCs is induced through a different pathway than the TLRs. However, CCL3 production was significantly reduced in TLR2^−/−^ and further reduced in MyD88^−/−^ DCs, although not abrogated (Fig. 3B). This indicates not only that CCL3 production by DCs is partially dependent upon TLR2 and MyD88 signaling but also that at least another pathway is involved in CPS-mediated activation of DCs. In addition, CCL2 and CCL3 production by CPS-stimulated WT DCs appears to be independent of the CPS structure, since the levels for both chemokines were all similar among samples from S. suis and S. pneumoniae (Fig. 3).

### *In vivo* immunogenicity studies with purified CPSs 3, 7, 8, and 9.

**FIG 4:**
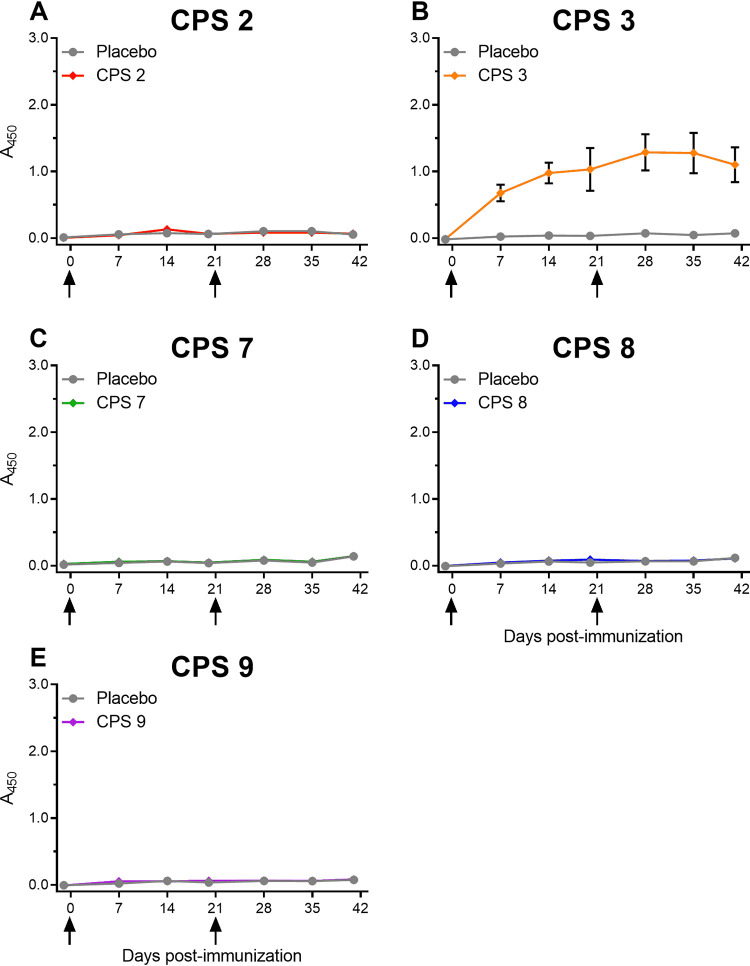
Kinetics of total antibody responses of mice immunized with 25 μg of either S. suis serotype 2 (A), serotype 3 (B), serotype 7 (C), serotype 8 (D), or serotype 9 (E) native purified CPS adjuvanted with TiterMax Gold. Mice (*n* = 10) were immunized on day 0 and boosted on day 21. Placebo mice (*n* = 10) were similarly injected with PBS adjuvanted with TiterMax Gold. ELISA plates were coated with native CPS from its respective serotype and incubated with blood samples diluted 1:100 to measure anti-CPS antibodies. Total Ig (IgG + IgM) antibody levels are shown as means ± the SEM of absorbance values at 450 nm. Placebo groups are indicated by a gray circle, whereas the CPS-immunized groups are indicated by a colored diamond. The arrow at day 21 indicates the boost.

Despite the absence of cytokine production and the lack of differences in chemokine production patterns after stimulation of APCs with widely different CPSs, the immunogenicity of those newly obtained purified CPSs was evaluated *in vivo*. Native purified CPS 2 (included as control) or native CPSs 3, 7, 8, and 9 were emulsified with TiterMax Gold and administered to mice on days 0 and 21. CPSs 2, 7, 8, and 9 failed to induce a significant anti-CPS response (Fig. 4). Although CPS 2 is already known to be poorly immunogenic, additional control enzyme-linked immunosorbent assays (ELISAs) were performed for CPSs 7, 8, and 9 to ensure that the lack of anti-CPS response was not due to problems with the coating (see Fig. S1 in the supplemental material). First, ELISA plates coated with whole S. suis of either serotype 7, 8, or 9 were used to validate strong total Ig (IgG + IgM) anti-S. suis responses in sera from hyperimmunized mice (Fig. S1A to C). Second, these control hyperimmune mouse sera showed no anti-CPS response for serotypes 7 and 8 (see Fig. S1D and E) but an anti-CPS response for serotype 9 (see Fig. S1F) using CPS-coated plates. Finally, sera collected at day 42 from mice immunized with purified CPSs 7, 8, and 9 showed no significant total Ig (IgG + IgM) anti-S. suis responses (*P* > 0.05 versus placebo groups) using whole-bacterium-coated plates. The latter result further supports that these CPSs did not elicit antibodies in the immunized animals and that this lack of response was not related to coating technical issues (see Fig. S1G to I). Taken together, these data indicate that purified CPSs 7, 8, and 9 are not immunogenic in mice.

**FIG 5:**
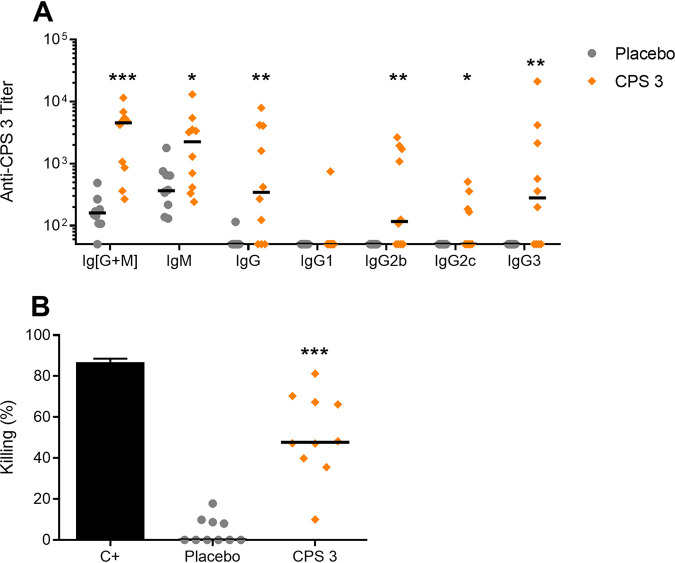
Isotyping and functional studies of antibodies produced in mice immunized with native S. suis serotype 3 purified capsular polysaccharide (CPS 3) in TiterMax Gold. Mice (*n* = 10) were immunized on day 0 and boosted on day 21 with 25 μg of CPS 3. Placebo mice (*n* = 10) were similarly injected with PBS adjuvanted with TiterMax Gold. Sera were collected on day 42. (A) For titration of anti-CPS antibody isotypes, ELISA plates were coated with native CPS 3 and incubated with 2-fold serial dilutions of sera, and isotypes were detected using specific HRP-conjugated anti-mouse total Ig [IgG + IgM], IgM, IgG, IgG1, IgG2b, IgG2c, or IgG3 antibodies. The results are expressed as titers for individual mice, with horizontal bars representing median. (B) Opsonophagocytosis killing of S. suis type 3 strain 4961 by day 42 sera from mice immunized with 25 μg of CPS 3 adjuvanted with TiterMax Gold. A 40% (vol/vol) sample serum and a bacterial MOI of 0.1 were added to fresh whole blood from naive mice to perform the assay. Viable bacterial counts were determined after 2 h of incubation. To determine bacterial killing, viable bacterial counts from tubes incubated with sample sera were compared to those incubated with control naive mouse sera. Rabbit anti-S. suis type 3 strain 4961 serum was used as a positive control (C+) and compared to control naive rabbit serum to determine bacterial killing. The results are expressed as percent bacterial killing for individual mice, with horizontal bars representing median. Individuals from the placebo group are indicated by a gray circle, whereas those of the CPS 3 group are indicated by an orange diamond. Statistically significant differences versus the placebo group are indicated (*, *P ≤ *0.05; **, *P ≤ *0.01; ***, *P ≤ *0.001).

In contrast, mice immunized with the native purified CPS 3 produced a strong antibody response as early as 7 days after a single dose, followed by a slight increase in the response after a boost on day 21 (Fig. 4B). A stronger response following boosting but also antibody isotype switching are good indicators of immunogenicity. Therefore, titers of the different anti-CPS antibody isotypes were determined in mice immunized with 25 μg of CPS 3 adjuvanted with TiterMax Gold. As shown in Fig. 5A, not only strong IgM titers but also significant high levels of type 1 IgG subclasses (IgG2b, IgG2c, and IgG3) were observed (*P ≤ *0.05 versus the placebo group), whereas no type 2 IgG subclass (IgG1) was detected (*P* > 0.05 versus the placebo group). Based on these observations, the protective capacity of sera from CPS 3-immunized mice was evaluated using an opsonophagocytosis assay (OPA), a recognized correlate of immunity for encapsulated Gram-positive bacteria (38). As shown in Fig. 5B, sera from mice immunized with CPS 3 adjuvanted with TiterMax Gold induced high bacterial killing levels (*P ≤ *0.001 versus placebo group).

### Adjuvant effect on *in vivo* immunogenicity of purified CPS 3.

The results showed that mice receiving on days 0 and 21 the same dose as before (25 μg) of CPS 3 either non-adjuvanted (phosphate-buffered saline [PBS] only) or adjuvanted 1:1 (vol/vol) with Alhydrogel 2% (39, 40) or 20 μg of Quil-A (41, 42) are unable to elicit anti-CPS 3 antibodies (see Fig. S2). Thus, purified CPS 3 is immunogenic in mice when adjuvanted with TiterMax Gold, leading to an opsonizing IgG antibody response.

### Purified CPS 3 immunogenicity is not influenced by phenol-extractable immune-stimulating ligands.

Since we showed that CPS 3 induces a type 1 IgG antibody response *in vivo*, we decided to investigate the presence of potential TLR ligands in the native CPS 3 preparation. As shown above, no native S. suis CPS, CPS 3 included, led to the production of IL-6 or TNF by CPS-stimulated DCs, in contrast to the cytokine production observed with S. pneumoniae CPS 19F (Fig. 2). In our study, the production of both IL-6 and TNF was dependent upon MyD88 signaling but independent of TLR2, suggesting that the CPS 19F preparation used contains immune-stimulating TLR ligands (see Fig. S3). After phenol extraction, S. suis CPS 3 did not show structural alterations by dot blot and by ^1^H nuclear magnetic resonance (NMR) analyses (see Fig. S4 and S5). DCs stimulated with phenol-extracted CPS 3 secreted no IL-6 nor TNF in the supernatant, and their CCL2 and CCL3 production remained unchanged compared to its native counterpart (data not shown).

**FIG 6:**
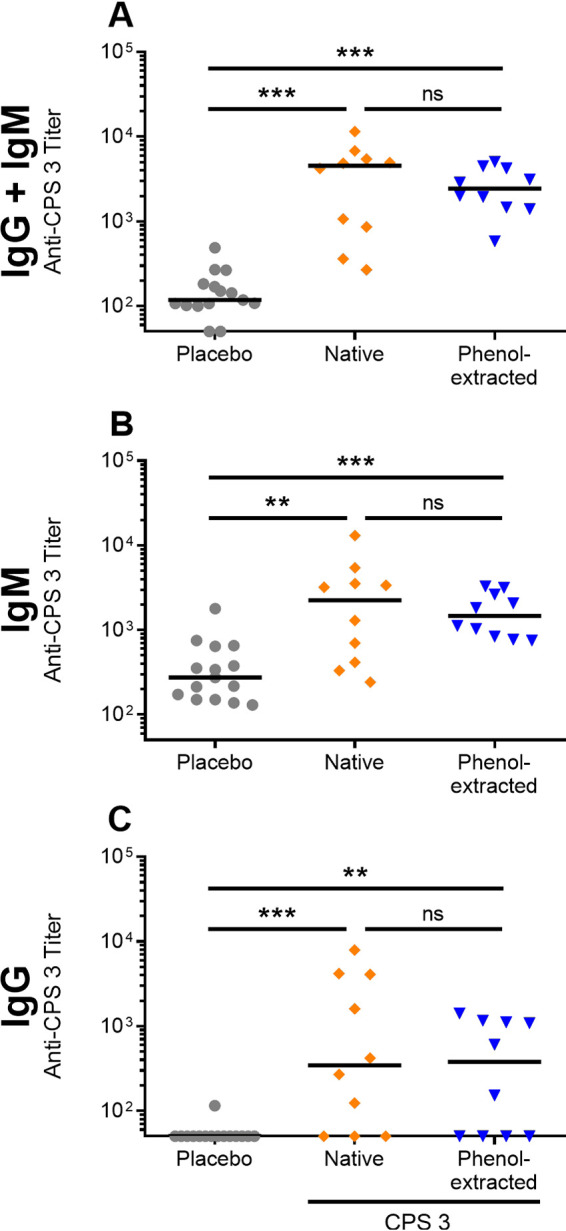
Isotyping of antibodies produced in mice immunized with phenol-extracted S. suis serotype 3 purified capsular polysaccharide (CPS 3) in TiterMax Gold. Mice (*n* = 10) were immunized on day 0 and boosted on day 21 with 25 μg of either native or phenol-extracted CPS 3. Placebo mice (*n* = 15) were similarly injected with PBS adjuvanted with TiterMax Gold. Sera were collected on day 42. For titration of anti-CPS antibody isotypes, ELISA plates were coated with native CPS 3 and incubated with 2-fold serial dilutions of sera, and isotypes were detected using specific HRP-conjugated anti-mouse total Ig (IgG + IgM), IgM, or IgG antibodies. The results are expressed as titers for individual mice, with horizontal bars representing the medians. Individuals from the placebo group are indicated by a gray circle, those of the native CPS 3 group are indicated by an orange diamond, and those of the phenol-extracted group are indicated by a blue triangle. Statistically significant differences between groups are indicated (ns, *P* > 0.05; **, *P ≤ *0.01; ***, *P ≤ *0.001).

To further determine whether potential TLR ligands affected the antibody response *in vivo*, mice received on days 0 and 21 a dose of 25 μg of either native or phenol-extracted CPS 3 adjuvanted with TiterMax Gold. Kinetic analysis of total Ig (IgG + IgM) antibody responses against native CPS 3 at days −1, 20, and 41 showed no significant differences (*P* > 0.05) between mice immunized with native and phenol-extracted CPS 3 (data not shown). Similarly, no significant differences were observed in anti-CPS 3 antibody isotypes (total Ig, IgM, and IgG) titers in sera collected on day 42 (*P* > 0.05; Fig. 6). These results suggest that immunogenicity of purified native CPS 3 *in vivo* is independent from phenol-extractable immune-stimulating ligands.

### *In vivo* anti-CPS responses to heat-killed encapsulated intact *S. suis* serotypes 2, 3, and 9.

**FIG 7:**
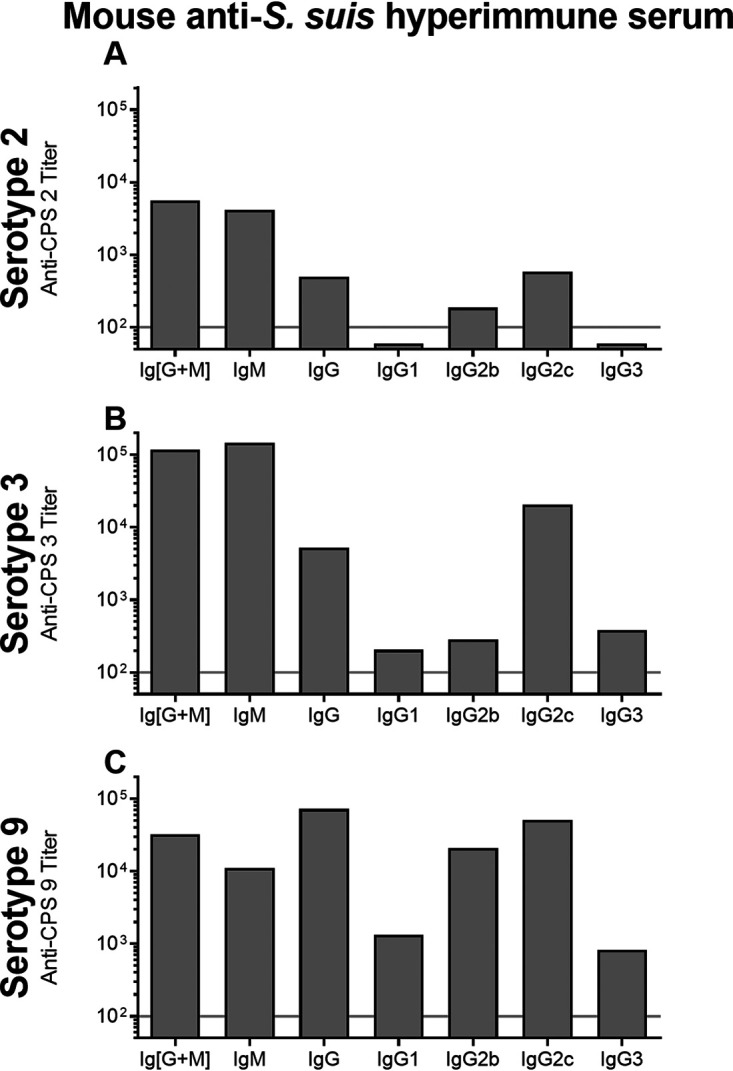
Isotyping of antibodies induced in control mice hyperimmunized with heat-killed S. suis serotype 2 strain S735 (A), serotype 3 strain 4961 (B), and serotype 9 strain 1273590 (C). Hyperimmune mice (*n* = 3 to 4) were obtained by repeated immunization with 1 × 10^9^ CFU/ml heat-killed S. suis by intraperitoneal injection weekly during 4 to 8 weeks. 2 weeks after the last injection, sera were collected and pooled by serotype. For titration of anti-CPS antibody isotypes, ELISA plates were coated with native CPS 2, CPS 3, or CPS 9 and incubated with 2-fold serial dilutions of sera, and isotypes were detected using specific HRP-conjugated anti-mouse total Ig (IgG + IgM), IgM, IgG, IgG1, IgG2b, IgG2c, or IgG3 antibodies. The results are expressed as titer, and the horizontal line behind the bars represents the cutoff; values found below are considered negative (titer < 100).

Following the previous immunizations performed using soluble antigens, we also investigated the CPS-specific antibody response in the context of encapsulated intact bacteria. The titers of the different anti-CPS antibody isotypes were determined in mice that were hyperimmunized by multiple weekly intraperitoneal injections with 1 × 10^9^ CFU of heat-killed S. suis. Since mice hyperimmunized with S. suis serotypes 7 and 8 showed no anti-CPS responses (see Fig. S1D and E), these two serotypes were not further studied. Pooled serum from mice hyperimmunized with S. suis serotype 2 showed intermediate levels of IgM and low levels of IgG that are specific to CPS 2, with barely detectable levels of IgG2b and low levels of IgG2c as the only IgG subclasses detected (Fig. 7A). In contrast, pooled serum from mice hyperimmunized with S. suis serotype 3 possessed high levels of IgM and intermediate levels of IgG specific to CPS 3 (Fig. 7B). Analysis of the anti-CPS 3 IgG subclasses showed high levels of IgG2c in contrast to barely detectable to low levels of IgG1, IgG2b, and IgG3. Finally, pooled serum from mice hyperimmunized with S. suis serotype 9 possessed intermediate levels of IgM and strong levels of IgG specific to CPS 9 (Fig. 7C). Analysis of the anti-CPS 9 IgG subclasses revealed high levels of IgG2b and IgG2c, in addition to low levels of IgG1 and IgG3. For all three serotypes, IgG2c was the dominant IgG subclass induced in hyperimmunized mice (Fig. 7).

### *In vivo* anti-CPS responses to live encapsulated intact *S. suis* serotypes 2, 3, and 9.

**FIG 8:**
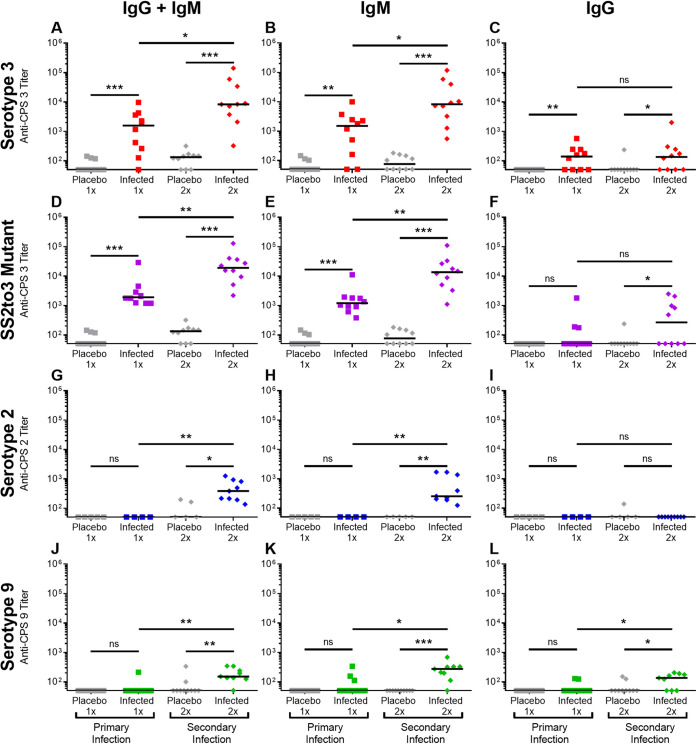
Anti-CPS antibody response after infections of mice with sublethal doses of S. suis serotype 3 strain 4961 (1 × 10^6^ CFU; A to C), a CPS-switched SS2to3 mutant of strain P1/7 (1 × 10^6^ CFU; D to F), serotype 2 strain P1/7 (1 × 10^6^ CFU; G to I), and serotype 9 strain 1135776 (1 × 10^5^ CFU; J to L). For the primary infection (1×), mice were infected on day 0, followed by serum collection on day 21. For the secondary infection (2×), mice were infected on day 0, reinfected on day 21, then sera were collected on day 42. Placebo mice were similarly injected once or twice with the vehicle solution (sterile Todd-Hewitt broth). Mouse groups were as follows: mice infected 1× or 2× with the serotype 3 and SS2to3 mutant and corresponding placebo mice (*n* = 10); mice infected with the serotype 2 (1×, *n* = 4; 2×, *n* = 9) and corresponding placebo mice (*n* = 5); mice infected with the serotype 9 (1×, *n* = 10; 2×, *n* = 9) and corresponding placebo mice (*n* = 10). For titration of anti-CPS antibody isotypes, ELISA plates were coated with serotype-matched native CPS and incubated with 2-fold serial dilutions of sera, and isotypes were detected using specific HRP-conjugated anti-mouse total Ig (IgG + IgM), IgM, or IgG antibodies. The results are expressed as titer for individual mice, with horizontal bars representing the medians. Individuals from the placebo groups are indicated by a gray symbol, whereas those of the infected groups are indicated by a colored symbol. Statistically significant differences between groups are indicated (ns, *P* > 0.05; *, *P ≤ *0.05; **, *P ≤ *0.01; ***, *P ≤ *0.001).

While titration of sera from mice hyperimmunized with heat-killed S. suis provided interesting data, we also studied the CPS-specific antibody response following infections with live S. suis, since this is the most relevant model. In the present study, a sublethal infection model was used to better characterize the antibody responses in the absence of significant inflammation and severe systemic clinical signs. During pretrials, different bacterial doses were tested, and sublethal doses (producing little to no mortality and/or severe clinical signs of disease) were chosen for each strain (data not shown). Titration of the different anti-CPS antibody isotypes after primary infection with S. suis serotype 3 revealed strong levels of IgM and low levels of IgG (*P ≤ *0.01) in comparison to placebo mice (Fig. 8A to C). A secondary infection with this serotype showed a significant boost for the IgM (*P ≤ *0.05) but not for the IgG (*P* > 0.05) response. In contrast to serotype 3, mice infected with S. suis serotype 2 showed no anti-CPS antibody titers following a primary infection, whereas a secondary infection induced low levels of IgM only (*P ≤ *0.01) (Fig. 8G to I). Sera from mice infected with S. suis serotype 9 displayed low levels of IgM and IgG that were not significant (*P* > 0.05) in comparison to placebo mice after a primary infection but nonetheless were boosted after a secondary infection (*P* > 0.05) (Fig. 8J to L). As such, while sublethal infections of mice with S. suis serotypes 2 and 9 induce low IgM anti-CPS responses only after a secondary infection, a sublethal infection with S. suis serotype 3 induce a high primary production of anti-CPS IgM which was further boosted after a secondary infection. Anti-CPS IgG responses were only observed for serotypes 3 (as a primary response) and 9 (as a secondary response).

### CPS 3 immunogenicity following sublethal infections is not influenced by the bacterial subcapsular domain.

Since we demonstrated that CPS 3 is immunogenic by itself (as a soluble purified antigen) but also when associated with the bacterial body (as a particulate antigen), we also opted to evaluate whether the immunogenicity of CPS 3 could be influenced by the composition and/or architecture of the bacterial subcapsular domain. Toward this aim, we used a CPS-switched SS2to3 mutant derived from the same S. suis serotype 2 strain previously used for the sublethal infections.

Titration of the different anti-CPS antibody isotypes in sera from mice that were sublethally infected with the SS2to3 mutant revealed strong levels of IgM (*P ≤ *0.001) and no significant levels of IgG (*P* > 0.05) after a primary infection in comparison to placebo (Fig. 8D to F). After a secondary infection with the SS2to3 mutant, a significant boost for the IgM (*P ≤ *0.05) and a significant IgG response (*P ≤ *0.05) were observed. Comparison of IgM and IgG responses in mice sublethally infected with either S. suis serotype 3 or the SS2to3 mutant after primary and secondary infections showed no significant difference between the two strains (*P* > 0.05), although the primary IgG response was lower in mice infected with the SS2to3 mutant (Fig. 8C and F). As such, the immunogenicity of CPS 3 associated with the whole bacteria appears to be independent from the bacterial subcapsular domain.

## DISCUSSION

The original idea behind this study stems from the fact that CPSs 2 and 14 of S. suis are particularly nonimmunogenic, even when associated at the bacterial surface in the course of a primary or booster experimental infection (12, 26, 27, 43–45). This low immunogenicity results in undetectable (CPS 14) to low IgM (CPS 2) titers and undetectable IgG titers for both CPSs (26, 27). Also, this low immunogenicity cannot just be explained by the presence of sialic acid, known to possess immunomodulatory properties (25, 27, 46). Following recent reports by our team of new repeating unit structures for S. suis CPSs, especially serotypes 3, 7, 8, and 9, we decided to further investigate the potential immunogenicity of these serotypes, as they are structurally well diversified among themselves and from the previously described sialylated serotypes.

Purified polysaccharides are known to be TI antigens and thus are generally poorly immunogenic. By inducing multivalent B cell receptor (mIg) cross-linking, TI antigens can induce proliferation of B cells and limited IgM responses lacking isotype switching. However, in the presence of a second signal such as TLR signaling, B cells activated by TI antigens undergo isotype switching and secrete substantial amounts of Ig (15). A previous study by Sen et al. has demonstrated that pneumococcal polysaccharide preparations used for vaccine production contain TLR2 and TLR4 ligands and that these TLR ligands substantially enhance primary and secondary antipneumococcal polysaccharide (PPS) responses in mice, especially the type 1 IgG subclasses (21). As such, those unappreciated copurified and/or contaminating TLR ligands in PPS vaccine preparations play an important role in providing the second signal for the induction of an *in vivo* IgG TI humoral response to purified polysaccharides.

Despite the importance of CPS as protective antigens and their effect on the development of antibody responses, few studies have been dedicated to the characterization of CPS activity on APCs and to the corresponding signaling mechanisms. Moreover, of those that are available, one must be cautious, since many of these studies describing innate immune activity of various purified polysaccharides have failed to rigorously rule out contaminating immune-stimulating ligands (15). It would be logical to assume that structural features of CPSs, such as different repeating unit compositions or glycosidic linkages, are susceptible to producing different immune responses, although such mechanisms remain largely unknown. Our results demonstrated that S. suis purified CPSs of serotypes 1, 1/2, 3, 7, 8, and 9 do not induce the release of key proinflammatory cytokines, which confirms their poor stimulatory activity. However, all these CPSs, including S. pneumoniae CPS 19F, stimulated DC production of two members of the CC family of chemokines, i.e., CCL2 and CCL3. These two chemokines are known to play a major role in the selective recruitment of monocytes, macrophages, DCs, and lymphocytes to sites of inflammation and also known to be produced in the course of S. suis infections (25, 47, 48). After stimulation with purified CPSs of S. suis and S. pneumoniae, DC production of CCL2 was shown to be independent from TLR2 and MyD88 pathways, whereas CCL3 production was partially dependent upon both TLR2 and MyD88. These observations are in agreement with previous results obtained using purified CPSs of S. suis serotypes 2 and 14 and of GBS serotypes III and V (25), suggesting that DCs appear to respond to CPSs in a patterned manner, seemingly independent from their different structural features. This warrants further investigations into the involvement of members from the large family of lectin receptors as possible candidates (49–52).

Mouse immunizations demonstrated that purified CPS 3 when adjuvanted with TiterMax Gold, a water-in-oil emulsion, induces the development of strong opsonizing type 1 IgG (mainly IgG2b and IgG3) and IgM antibody responses, in stark contrast to CPSs 2, 7, 8, and 9 (this study) and CPS 14 (27). Immunization of purified CPS 3 alone or combined with Alhydrogel or with Quil-A as adjuvants failed to mount an antibody response. Nonetheless, this makes CPS 3 as the first immunogenic purified CPS of S. suis. The current model for polysaccharide-specific TI humoral immunity, as proposed by Snapper (15), requires two signals to ensure antibody secretion and class switching. Signal 1 is delivered through multivalent B cell receptor cross-linking by polymeric TI antigens. Signal 2 is delivered to CPS-specific B cells by innate cells through cytokine (such as IFN-γ, granulocyte-macrophage colony-stimulating factor, and IL-3) and/or BAFF/APRIL production, which are induced in response to PAMPs (such as TLR ligands). However, we showed that our purified CPS preparations are exempt from phenol-extractable contaminants and from nucleic acids and proteins and also that they fail to induce cytokine production by DCs, at least those evaluated in our study. Another possible source for signal 2 *in vivo* could be from the adjuvants used. A previous study has shown that stimulation of porcine DCs with TiterMax Gold with no antigen induced IL-6 production, whereas stimulation with either Alhydrogel 2% or Quil-A induced IL-6 and/or IL-12 production, suggesting that these adjuvants by themselves can trigger signal 2 following immunization with purified CPSs (53). Yet, only the combination of CPS 3 with TiterMax Gold induced a potent protective response. This could be explained by the particulate form of the water-in-oil emulsion. Relative to soluble antigens, antigens in particulate form are phagocytosed more efficiently, produce higher levels of immune activation, and deliver concentrated antigens per particle than could be achieved through pinocytosis of soluble antigens (54, 55). Of note, aluminum salt-based adjuvants, such as Alhydrogel 2%, are themselves particulate, but antigens adsorbed to them do not behave as particulates (54, 55). Nevertheless, the adjuvant properties of TiterMax Gold do not benefit to the immunogenicity of other CPSs studied. Taken together, our results suggest that purified CPS 3 possesses unique structural and/or biochemical properties. In this regard, it was observed that water soluble CPS 3 was precipitated in the presence of the detergent sodium deoxycholate, suggesting an amphiphilic character for this polysaccharide (56). The hydrophobic element is most likely the di-*N*-acetylated d-bacillosamine, as acetylation increases hydrophobicity (such as is the case for chitin versus chitosan [57]), which is then linked to the negatively charged d-glucuronic acid, together forming the amphiphilic disaccharide repeating unit of CPS 3 (Fig. 1). In turn, this amphiphile could be copresented at the water-oil interface and/or associate with the amphiphilic block copolymer component of the TiterMax Gold emulsion in a similar fashion to its presentation at bacterial surfaces, and fully benefit from its adjuvant properties (58). Nonetheless, further studies into the structural and biochemical properties of purified CPS 3 and their effect on the induction of humoral immunity are warranted.

The immunogenicity of CPS 3 was also demonstrated in mice injected with serotype 3 intact whole bacteria, either heat killed via hyperimmunization or live via sublethal infections. Mice infected/immunized with the serotype 3 bacteria produced a very robust IgM anti-CPS antibody response in addition to isotype-switched IgGs. In addition, and in contrast to soluble purified CPS, a very strong anti-CPS 9 IgG response was observed in mice hyperimmunized with heat-killed serotype 9, whereas mice sublethally infected showed a weak IgM and IgG response only after a secondary infection. Due to the virulence of the serotype 9 strain used for sublethal infections, lower doses had to be used, which might explain the lower response observed. This displayed immunogenicity for CPSs 3 and 9 in the context of whole intact bacteria is in agreement with the previously mentioned model for polysaccharide-specific TI humoral immunity: as CPSs are displayed at the bacterial surfaces, there are plenty of bacterial PAMPs, such as TLR2 ligands, to trigger signal 2 by APCs leading to stronger antibody secretion and isotype switching (15). Differences in these signals might also explain divergences in IgG switching, IgG subclasses, and/or presence/amplitude of memory (boost) anti-CPS responses between serotypes. Immunization/infection with intact serotype 2 whole bacteria led to the lowest anti-CPS response (in quantity and quality). However, a recipient serotype 2 strain displaying a serotype 3 CPS induced optimal anti-CPS 3 antibody response, confirming the poorly immunogenic nature of CPS 2 and that anti-CPS 3 response to whole bacteria is independent from the bacterial subcapsular domain. A previous study has reported an important role of bacterial subcapsular domain in the anti-CPS response against S. pneumoniae serotype 14 by studying an asialo mutant of GBS serotype III, which expresses an identical CPS (59). The absence of this effect in our model could be due to the fact that intraspecies serotype switching was used to better reflect serotype divergences within the S. suis bacterium.

Even though the predominant IgG subclasses differ between purified CPS and intact bacteria for serotype 3, or among serotypes for hyperimmunized mice, the anti-CPS response remains biased toward the type 1 subclasses of IgG (IgG2a/IgG2c, IgG2b, and IgG3), which are known to be superior in both opsonophagocytosis activity and complement activation than the type 2 (IgG1) subclass (60, 61). Also, sublethal infections with serotypes 2, 3, and 9 led to boosted IgM and/or IgG titers following a secondary infection, a feature of T cell-dependent responses, in contrast to the TI response to purified CPS 3, which failed to show a significant boost following the second dose. Taken together, these observations demonstrate a number of consistent differences between intact bacteria in general versus purified CPSs, suggesting that different mechanisms are involved in the development of anti-CPS humoral responses (15, 59, 62–64).

CPS immunogenicity might differ between species. For example, rabbits are used to generate the anti-CPS specific antisera used by diagnostic services to perform S. suis serotyping (65). Therefore, rabbits are able to generate anti-CPS antibodies to all known S. suis serotypes after hyperimmunization (66–69). Nevertheless, our findings with the mouse model seem to correlate with the scarcely available data on CPS immunogenicity in the natural host, the pig. Some studies have demonstrated the generation of anti-CPS 2 antibodies in swine, albeit at very low levels, depending on the model used (12, 44, 45, 70, 71). Indeed, most studies were performed by either (i) immunization with whole killed bacteria or with CPS-conjugate preparations or (ii) using either natural infection (i.e., samples from the field) or experimental infection. One study has shown that purified CPS 2 can induce anti-CPS 2 specific antibodies in swine when using Freund's complete adjuvant (43). A single study has reported low levels of anti-CPS 7 in naturally exposed piglets (72). Therefore, swine seems to have a B cell repertoire able to recognize, at least, CPSs 2 and 7. Nevertheless, as shown in mice, immunogenicity is low unless immunization strategies such as CPS conjugates, strong adjuvants, or hyperimmunization are used. Future studies on swine anti-CPS antibody generation are warranted.

The results obtained in this study using either purified CPSs or whole bacteria have strong implications for the design of effective vaccines against S. suis and provide novel insights into the complexity of the anti-CPS response. Indeed, we have previously demonstrated that a serotype 2 glycoconjugate vaccine is able to induce a protective response in mice and pigs (12), thus highlighting the importance of CPS as a vaccine target. Consequently, understanding the development of the anti-CPS response to different S. suis serotypes is of utmost importance for vaccine development against this economically important pathogen and zoonotic agent. Our data further support the importance of the Th1 bias and/or the type 1 antibody response in the immunopathogenesis of S. suis infections and development of a protective response. Finally, this study provides important information on the divergent evolution of CPS serotypes with highly different structural and/or biochemical properties within S. suis and their interaction with the immune system.

## MATERIALS AND METHODS

### Bacterial strains and culture conditions.

Bacterial strains that were used in this study are listed in Table 1. Isolated colonies on sheep blood agar plates were inoculated in 5 ml of Todd-Hewitt broth (THB; Oxoid, Nepean, Ontario, Canada) and incubated for 8 h in a water bath at 37°C with agitation at 120 rpm. Working cultures were prepared by transferring 10 μl of 8-h cultures diluted 1:1,000 with phosphate-buffered saline (PBS) into 30 ml of THB, which was incubated for 16 h. Bacteria were washed three times and resuspended in PBS to obtain a concentration between 1 × 10^8^ to 1 × 10^9^ CFU/ml. Heat-killed bacterial cultures were obtained as previously described (73). Briefly, overnight cultures were washed three times with PBS and then resuspended in 30 ml of fresh PBS. A sample was taken to perform bacterial counts on Todd-Hewitt agar (THA). Bacteria were immediately killed by incubating at 60°C for 45 min and then cooled on ice. Bacterial killing was confirmed by absence of growth on blood agar for 48 h.

A CPS-switched 2-to-3 mutant (SS2to3) derived from the S. suis serotype 2 strain P1/7 was also used for the sublethal infections (Table 1). The first step in the creation of this mutant consists of the genetic excision in this receiver strain of the whole *cps-2* locus genes (responsible for CPS expression). For this, a DNA fragment comprising a chloramphenicol resistance gene (*cat*) cassette flanked by ∼1 kbp of the upstream and downstream regions of the *cps-2* gene cluster (in the receiver strain) was amplified by overlap-extension PCR. The receiver strain was transformed with the *cat* fragment to yield a nonencapsulated mutant (ΔCPS2). Then, the receiving ΔCPS2 mutant strain was transformed with a whole genome of a donor S. suis serotype 3 strain to yield the desired serotype-switched mutant SS2to3 through the replacement of the *cat* fragment with the *cps-3* locus (M. Okura, unpublished data). Verification of this mutant by whole-genome sequencing (accession no. WABX00000000), transmission electron microscopy, serology, and NMR analyses of its purified CPS all confirmed its phenotype as a CPS 3-expressing well-encapsulated serotype-switched mutant (M. Okura, unpublished data). While expressing CPS 3 as its capsule, the subcapsular antigens of this mutant are from a phenotypically very different serotype 2 strain.

### *S. suis* capsular polysaccharides: production and purification.

The following methods for CPS production and purification were adapted from previous publications (23, 28–31). Briefly, 6 liters of fresh THB was inoculated with the appropriate strain and incubated overnight. For more information on the strains used for CPS production, refer to Table 1.

Depending on the sensitivity of the specific CPS to hydrolysis, two methods of CPS extraction were used. For serotypes 2, 1, 1/2, and 3, the autoclave method was used (23, 28, 30). Briefly, bacterial cells from the 6-liter culture were pelleted by centrifugation at 10,000 × *g* for 40 min, suspended by repeated pipetting in a buffer containing 33 mM phosphate and 145 mM NaCl (pH 8.0), and chilled. The bacterial suspension was autoclaved at 121°C for 15 min. The supernatant containing the crude CPS was recovered by centrifugation at 9,000 × *g* for 50 min. For serotypes 7, 8, and 9, the water method was used (29–31). Briefly, the bacterial pellet from the 6-liter culture was resuspended in deionized water (ddH_2_O). The bacteria were killed by heating at 60°C for 45 min, as the standardized protocol for S. suis. The content of the tubes was freeze-dried for 72 h. Then, bacterial cells were stirred with ddH_2_O (3 g of dry cells in 100 ml) overnight at room temperature. The supernatant containing the crude CPS was recovered by centrifugation at 9,000 × *g* for 50 min.

The crude CPSs were further purified from the extracted material by solvent extraction with chloroform to remove lipids, precipitation with 25% (vol/vol) ethanol and 0.1 M CaCl_2_ to remove nucleic acids, precipitation of CPS and other macromolecules with 80% (vol/vol) ethanol, dialysis, and freeze-drying. The final purification step to remove proteins was performed by gel filtration chromatography as previously described (30). Briefly, an XK-26/100 column packed with Sephacryl S-400 HR (GE Healthcare Life Sciences, Uppsala, Sweden) was used and eluted with 50 mM NH_4_HCO_3_ (for serotypes 2, 1, 1/2, and 3) or with 50 mM NaCl (for serotypes 7, 8, and 9) at a flow rate of 1.3 ml/min, using an ÄKTA Purifier 10 system (GE Healthcare Life Sciences), including a UV-900 monitor, and equipped with a Knauer Smartline 2300 RI detector (Knauer, Berlin, Germany) connected to the system via an AD-900 analog/digital converter (GE Healthcare Life Sciences). Fractions giving a positive RI signal but no absorption at 280 and 254 nm were pooled and freeze-dried. Dot blot confirmation of CPS-containing fractions with reference antisera has already been performed previously (23, 28–31). To desalt, the purified material was dissolved in water, dialyzed against ddH_2_O for 24 h at 4°C, and finally freeze-dried.

### *S. pneumoniae* capsular polysaccharide.

Purified pneumococcal polysaccharide of type 19F was manufactured by Pfizer and purchased through the American Type Culture Collection (ATCC; 84-X).

### CPS quality controls.

Each CPS was subjected to rigorous quality control tests as previously described (25) on 1-mg/ml samples. Nucleic acids were quantified using an ND 1000 spectrophotometer (NanoDrop, Wilmington, DE). The absorbance was measured at 230 and 260 nm on CPS samples. Calculations were done with the NanoDrop software. According to the manufacturer, results are reproducible between 2 and 100 ng/μl. Proteins were quantified on CPS samples using the modified Lowry protein assay kit (Thermo Fisher Scientific, Saint-Laurent, Quebec, Canada) in a 96-well microplate according to the manufacturer’s instructions. The calculated limit of detection (*P ≤ *0.05) was 5 μg/ml for the modified Lowry assay. Proteins were also quantified on CPS samples using a NanoOrange protein quantitation kit (Molecular Probes, Eugene, OR) according to manufacturer’s instructions by heating samples into microtubes, followed by transfer to a 96-well microplate for fluorescence reading. The calculated limit of detection (*P ≤ *0.05) was 4 μg/ml for the NanoOrange assay.

### Mouse strains.

Mice originating from Jackson Laboratory (Bar Harbor, ME), including WT C57BL/6J, MyD88^−/−^ [B6.129P2(SJL)-*Myd88*^tm1Defr^/J], and TLR2^−/−^ (B6.129-*Tlr2*^tmlKir^/J) mice, were used to generate bone marrow-derived DCs. C57BL/6 mice from Charles River (Wilmington, MA) were used to perform the immunizations and infections. All experiments involving mice were carried out in accordance with the recommendations of the guidelines and policies of the Canadian Council on Animal Care and the principles set forth in the *Guide for the Care and Use of Laboratory Animals*. The protocols and procedures were approved by the Animal Welfare Committee of the University of Montreal (protocols rech-1399 and rech-1523).

### Generation of bone marrow-derived DCs.

Bone marrow-derived DCs were produced according to a previously described technique (25, 74) and cultured in complete cell culture medium consisting of RPMI 1640 supplemented with 5% heat-inactivated fetal bovine serum, 10 mM HEPES, 2 mM l-glutamine, 50 μM 2-mercaptoethanol, 100 U/ml penicillin-streptomycin, and 20 μg/ml gentamicin. All reagents were from Gibco (Invitrogen, Burlington, Ontario, Canada). Cell purity was routinely at ≥86 to 90% CD11c^+high^ F4/80^-/dim^ cells as determined by fluorescence-activated cell sorter analysis (25, 75).

### *In vitro* DC stimulation assay.

DCs were resuspended at 10^6^ cells/ml in complete medium and stimulated with CPS (final concentration, 200 μg/ml) essentially as described in Calzas et al. (25). CPS samples were prepared from 2-mg/ml stock solutions and treated with polymyxin B sulfate (final concentration, 20 μg/ml; Sigma-Aldrich, Oakville, Ontario, Canada) 1 h prior to activation to neutralize any possible endotoxin contamination. After 24 h, supernatants were collected for cytokine quantification by ELISA. Cells stimulated with 1 μg/ml purified Escherichia coli O127:B8 lipopolysaccharide (LPS; Sigma-Aldrich) served as a positive control for cytokine production. Unstimulated cells served as a negative control. All solutions were tested for the absence of endotoxin by use of a *Limulus* amebocyte lysate gel-clotting test (Pyrotell; ACC, East Falmouth, MA).

### Cytokine quantification by ELISA.

Levels of IL-6, TNF, CCL2, and CCL3 in cell culture supernatants were measured by sandwich ELISAs using pair-matched antibodies from R&D Systems (Minneapolis, MN), performed according to the manufacturer’s recommendations.

### Mouse immunization studies.

Five-week-old C57BL/6 female mice were immunized subcutaneously with a dose of 25 μg of purified CPS in a volume of 0.1 ml on day 0 and boosted on day 21 as previously reported (12). In a first experiment aimed to evaluate the immunogenicity of the CPSs from the different serotypes, five groups of mice (*n* = 10) received 25 μg of either S. suis serotype 2 CPS (CPS 2), CPS 3, CPS 7, CPS 8, or CPS 9 dissolved in PBS and emulsified 1:1 (vol/vol) with TiterMax Gold (CytRx Corporation, Norcross, GA) as the adjuvant. One placebo group of mice (*n* = 10) received PBS adjuvanted with TiterMax Gold as described above. In a second experiment aimed to compare different adjuvants with CPS 3, three groups of mice (*n* = 10) received 25 μg of CPS 3 either dissolved in PBS (without adjuvant), or adjuvanted either with 1:1 (vol/vol) Alhydrogel 2% (Brenntag Biosector, Frederikssund, Denmark) or with 20 μg of Quil-A (Brenntag Biosector). Three placebo groups of mice (*n* = 10) were included: the first received PBS only (no adjuvant), the second received PBS adjuvanted with Alhydrogel 2%, and the third received PBS adjuvanted with Quil-A as described above. In a third experiment aimed to evaluate the immunogenicity of phenol-extracted CPS 3 (described below), two groups of mice (*n* = 10) received 25 μg of either native or phenol-extracted CPS 3 adjuvanted with TiterMax Gold (see above). One placebo group of mice (*n* = 5) which received PBS adjuvanted with TiterMax Gold was also included.

In the first experiment to follow the kinetics of the antibody responses, mice were bled (10 μl) weekly on days −1, 7, 14, 20, 28, 35, and 41 postimmunization by the tail vein. In the other two experiments, mice were bled (10 μl) on days −1, 20, and 41 postimmunization by the tail vein to monitor antibody responses. Diluted blood was directly used in the ELISA as described below. At day 42 postimmunization, mice were humanely euthanized, and sera were collected and frozen at −80°C for ELISA Ig titration and isotyping analyses and for opsonophagocytosis assay (see below).

### Control antisera: mouse hyperimmunization.

Hyperimmune mice (*n* = 3 to 4) were obtained by repeated immunization of 5-week-old female C57BL/6 mice with 1 × 10^9^ CFU/ml heat-killed S. suis in THB by intraperitoneal injection weekly during 4 to 8 weeks. For more information on the strains used for mouse hyperimmunization, refer to Table 1. At 2 weeks after the last injection, sera were collected, pooled by serotype, aliquoted, and stored at −80°C.

### Measurement of antibodies against *S. suis* CPSs and whole bacteria.

The following methods for coating of microplates and the measurement of antibodies by ELISA were taken or adapted from previous publications (12, 26, 76). For coating of ELISA plates with CPS 2, CPS 3, and CPS 9, CPSs were diluted to 2 μg/ml in 0.1 M NaHCO_3_ (pH 9.6); then, 100-μl portions were added to the wells of a 96-well Polysorp microplate (Nunc-Immuno; Canadawide Scientific, Toronto, Ontario, Canada). For coating with CPS 7 and CPS 8, the CPSs were diluted to 10 and 5 μg/ml, respectively, and then 100-μl portions were added to the wells of a 96-well EIA/RIA microplate (medium binding; Thermo Fisher Scientific). CPS-coated plates were left overnight at 4°C. For coating of ELISA plates with whole bacteria, bacterial cultures were prepared as described above and resuspended in ddH_2_O to an optical density at 600 nm (OD_600_) of 0.500 to 0.550, which corresponds to approximately 1 × 10^8^ CFU/ml. For more information on the strains used for ELISA plate coating, refer to Table 1. To the wells of a 96-well Polysorp microplate was added 100 μl of the bacterial suspension; this was left to air dry at room temperature for 48 h. Then, 50 μl of methanol was added to each well, and the samples were left to evaporate at room temperature for a few hours. When completely dry, the plates were stored at room temperature.

Plates were washed with PBS containing 0.05% (vol/vol) Tween 20 (PBST) and blocked by treatment with 300 μl of PBS containing 1% (wt/vol) bovine serum albumin (HyClone, Logan, UT) for 1 h. After washing, 100 μl of mouse blood or serum samples diluted in PBST was added to the wells, and the plates were left for 1 h. After washing, the plates were incubated for 1 h with 100 μl of a horseradish peroxidase (HRP)-conjugated isotype-specific antibody diluted in PBST as described below. The enzyme reaction was developed by addition of 100 μl of 3,3′,5,5′-tetramethylbenzidine (TMB; 100 μl) (Invitrogen) and stopped by the addition of 50 μl of 0.5 M H_2_SO_4_, and the absorbance was read at 450 nm with an ELISA plate reader.

To follow the kinetics of total Ig (IgG + IgM) antibody responses to CPS, mouse blood collected from the tail vein was diluted 1:100. Dilution optimization had previously been conducted (data not shown). HRP-conjugated goat anti-mouse total Ig (IgG + IgM) (H+L) at a dilution of 1:2,500 (Jackson ImmunoResearch, West Grove, PA) was used as a detection antibody.

To perform the titration of mouse Ig isotypes, day-42 serum was serially diluted (2-fold) in PBST, and antibodies were detected using either HRP-conjugated goat anti-mouse total Ig [IgG + IgM] as mentioned above, goat anti-IgG (Fcγ fragment specific; Jackson Immunoresearch), goat anti-IgM diluted 1:1000, goat anti-IgG1, goat anti-IgG2b, goat anti-IgG2c, or goat anti-IgG3 diluted 1:400 (Southern Biotech, Birmingham, AL). For mouse serum titration, the reciprocal of the last serum dilution that resulted in an OD_450_ of ≤0.2 (as a preestablished cutoff for comparison purposes) was considered the titer of that serum. For representation purposes, negative titers (less than or equal to the cutoff) were given an arbitrary titer value of 50.

To control interplate variations, an internal reference positive control was added to each plate. For titration of mouse antibodies, this control was a pool of sera from hyperimmunized mice (produced as described above). Reaction in TMB was stopped when an OD_450_ of 1.0 was obtained for the positive internal control. Optimal coating conditions for the anti-CPS and anti-S. suis ELISAs, optimal dilutions for the positive internal control sera, and the HRP-conjugated anti-mouse antibodies were determined during preliminary standardizations.

### Opsonophagocytosis assay for serotype 3.

Instead of using a cell line or a single cell type, the OPA was standardized using whole blood from naive mice (12, 77). This model considers all blood leukocytes and thus represents a more realistic model of the complex interactions between all immune cells and the bacteria during a systemic infection, as is the case for S. suis.

Murine whole blood OPAs were performed as previously described (12, 77). Blood was collected by intracardiac puncture from naive C57BL/6 mice, treated with sodium heparin, and then diluted to obtain 6.25 × 10^6^ leukocytes/ml in complete cell culture medium. All blood preparations were kept at room temperature. Using washed bacterial cultures of S. suis serotype 3 strain 4961 (Table 1) grown as described above, final bacterial suspensions were prepared in complete cell culture medium to obtain a concentration of 1.25 × 10^6^ CFU/ml. The number of CFU/ml in the final suspension was determined by plating samples onto THA. All bacterial preparations were kept on ice.

Diluted whole blood (5 × 10^5^ leukocytes) was mixed with 5 × 10^4^ CFU of S. suis (multiplicity of infection [MOI] of 0.1) and 40% (vol/vol) of sera from naive or immunized mice in a microtube to a final volume of 0.2 ml. The tube tops were pierced using a sterile 25-gauge needle, and then the microtubes were incubated for 2 h at 37°C with 5% CO_2_, with gentle manual agitation every 20 min. After incubation, viable bacterial counts were performed on THA. Tubes with addition of naive rabbit serum or rabbit anti-S. suis type 3 serum (65) were used as negative and positive controls, respectively. The bacterial killing percentage was determined by using the following formula: percentage of bacteria killed = [1 − (bacteria recovered from sample tubes/bacteria recovered from negative-control tubes with naive mouse sera)] × 100. Final OPA conditions were selected based on several pretrials using different incubation times and MOIs (data not shown).

### Phenol extraction of *S. suis* serotype 3 CPS.

Phenol extraction of CPS 3 was conducted as described by Sen et al. (21) with some modifications. Native CPS 3 was dissolved in water containing 0.2% triethanolamine (TEA) to a final CPS concentration of 5 mg/ml. Sodium deoxycholate was omitted, since it caused CPS 3 to precipitate. Water-saturated phenol (0.5 ml) was added, and the tube was vortexed intermittently for 5 min. The solution was left standing for 5 min at room temperature for phase separation, then was placed on ice for an additional 5 min. The material was centrifuged for 2 min at 10,000 × *g* at 4°C. The upper aqueous layer was transferred to another tube. The phenol layer was re-extracted with 0.5 ml of water containing 0.2% TEA. The aqueous layers were pooled and re-extracted with 1 ml of water-saturated phenol. Pooled aqueous layers were adjusted to 80% ethanol containing 30 mM sodium acetate and left overnight at 4°C to precipitate. The material was then centrifuged for 10 min at 10,000 × *g* at 4°C. The precipitate was washed with 1 ml of ice-cold absolute ethanol and then air-dried. CPS 3 was redissolved in 1 ml of water and freeze-dried to be weighed subsequently.

### Dot blotting.

Dot blot analyses were performed essentially as described previously by Van Calsteren et al. (28). Ten microliters (each at 1 mg/ml) of both native and phenol-extracted CPS 3 and of CPS 2 (used as negative control) were blotted on a polyvinylidene difluoride Western blot membrane (Bio-Rad, Hercules, CA). The membrane was blocked for 1 h with a solution of Tris-buffered saline (TBS) containing 2% skim milk, followed by 2 h of incubation with rabbit anti-S. suis type 3 serum. The membrane was washed three times with TBS, and anti-rabbit HRP-conjugated antibody (Jackson Immunoresearch) was added for 1 h. The membrane was washed three times with TBS and revealed with a 4-chloro-1-naphthol solution (Sigma-Aldrich).

### NMR spectroscopy.

Both native and phenol-extracted CPS 3 were exchanged in 33 mM phosphate buffer pD 8.0 in D_2_O (99.9 atom% D), freeze-dried, and dissolved in D_2_O (99.96 atom% D). NMR spectra were acquired on CPS samples at concentrations of 0.6 to 0.7% at 11.75 T using a Bruker Avance 500 spectrometer equipped with a 5-mm triple resonance TBI probe with ^1^H, ^13^C, and ^109^Ag–^31^P channels at 60°C using standard Bruker pulse sequences at the Centre Régional de Résonance Magnétique Nucléaire (Department of Chemistry, University of Montreal). For conventional ^1^H spectra, 32 K complex data points were acquired after a 30° pulse with a digital resolution of 0.18 Hz/point and processed off-line using SpinWorks v4.2.8.0 (Kirk Marat, home.cc.umanitoba.ca/~wolowiec/spinworks/) by exponential multiplication with a 0.2-Hz line broadening factor, zero filling, complex Fourier transform, phase correction, and fifth-order polynomial baseline correction. ^1^H chemical shifts δ in ppm were referenced to internal deuterated 2,2-dimethyl-2-silapentane-5-sulfonate at δ 0 as recommended by Wishart et al. (78).

### Experimental mouse sublethal infections.

Six-week-old female C57BL/6 were injected intraperitoneally with sublethal doses of the S. suis strains used (1 × 10^6^ CFU for serotype 2, serotype 3, and CPS-switched SS2to3 mutant; and 1 × 10^5^ CFU for serotype 9). For more information on the strains used for experimental infections, refer to Table 1. These sublethal doses were chosen since they produced mild clinical signs and low mortality in our infection model in order to follow the antibody response. For the primary infection model, groups of mice (*n* = 4 to 10) were infected on day 0 followed by serum collection on day 21. For the secondary infection model, groups of mice (*n* = 9 or 10) were infected on day 0, reinfected on day 21; sera were then collected on day 42. Placebo groups of mice (*n* = 5 to 10) were similarly injected once or twice with the vehicle solution (sterile THB). Infected animals were monitored at least twice daily for the first 7 days after each infection and then daily until the end of the study. Blood bacterial titers were assessed 24 h following the first injection on day 0 by collecting 5 μl of blood from the tail vein. Proper dilutions in PBS were plated onto THA.

### Statistical analyses.

Parametric data are expressed as means ± standard errors of the means (SEM) and were analyzed for significance using one-way analysis of variance (ANOVA) followed by the Tukey test. Nonparametric data are shown with the median and were analyzed for significance using either the Mann-Whitney Rank Sum Test (to compare two groups) or the Kruskal-Wallis ANOVA on ranks, followed by the Dunn procedure (to compare three groups or more). Statistical analyses were performed using Systat SigmaPlot v11.0.

## Supplementary Material

Supplemental file 1
