# Supplementary material for: Comparative Study of Immunogenic Properties of Purified Capsular Polysaccharides from Streptococcus suis Serotypes 3, 7, 8, and 9: the Serotype 3 Polysaccharide Induces an Opsonizing IgG Response
Source: Infect Immun. 2020 Sep 18;88(10):e00377-20. doi: 10.1128/IAI.00377-20 (PMC7504959; doi:10.1128/IAI.00377-20)
Supplement: Supplemental file 1 [file IAI.00377-20-s0001.pdf]

SUPPLEMENTARY MATERIAL

Comparative study of immunogenic properties of purified capsular polysaccharides from *Streptococcus suis* serotypes 3, 7, 8, and 9: the serotype 3 polysaccharide induces an opsonizing IgG response

Guillaume Goyette-Desjardins,[a,b,c] Jean-Philippe Auger,[a,b,c] Dominic Dolbec,[a,b,c] Evgeny Vinogradov,[d] Masatoshi Okura,[e] Daisuke Takamatsu,[e,f] Marie-Rose Van Calsteren,[a,g] Marcelo Gottschalk,[a,b,c] Mariela Segura[a,b,c]#

[a]Swine and Poultry Infectious Diseases Research Centre, Faculty of Veterinary Medicine, University of Montreal, Saint-Hyacinthe, Quebec, Canada
[b]Research Group on Infectious Diseases in Production Animals, Faculty of Veterinary Medicine, University of Montreal, Saint-Hyacinthe, Quebec, Canada
[c]Canadian Glycomics Network (GlycoNet), University of Alberta, Edmonton, Alberta, Canada
[d]National Research Council, Ottawa, Ontario, Canada
[e]Division of Bacterial and Parasitic Disease, National Institute of Animal Health, National Agriculture and Food Research Organization, Tsukuba, Ibaraki, Japan
[f]The United Graduate School of Veterinary Sciences, Gifu University, Gifu, Gifu, Japan
[g]Saint-Hyacinthe Research and Development Centre, Agriculture and Agri-Food Canada, Saint-Hyacinthe, Quebec, Canada

#Address correspondence to Mariela Segura, mariela.segura@umontreal.ca

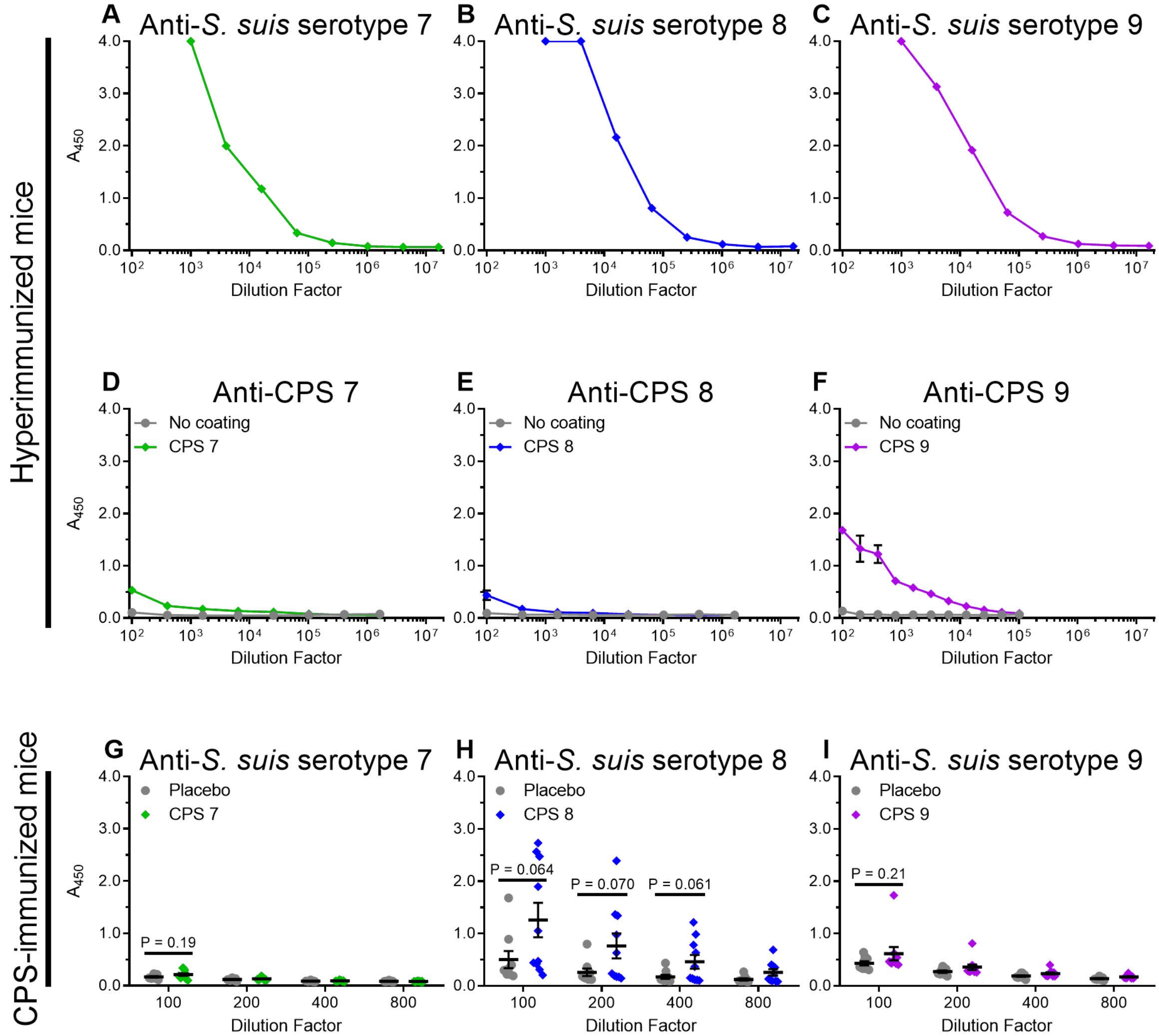

FIG S1 Additional controls for the total antibody response of mice immunized with purified CPSs of *S. suis* serotype 7 (CPS 7), serotype 8 (CPS 8), and serotype 9 (CPS 9) (see Fig. 5). (A–C) Total anti-*S. suis* response of control sera from hyperimmunized mice. Hyperimmune mice (*N* = 3–4) were obtained by repeated immunization with 1 × 10^9 CFU/ml heat-killed *S. suis* by intraperitoneal injection weekly during 4 to 8 weeks. Two weeks after the last injection, sera were collected and pooled by serotype. ELISA plates were coated with whole bacteria of either *S. suis* serotype 7 strain 1750775 (A), serotype 8 strain 1719887 (B), or serotype 9 strain 1273590 (C) and

incubated with serial dilutions of sera from hyperimmunized mice. Antibodies were detected using specific HRP-conjugated anti-mouse total Ig [IgG + IgM]. Results are shown as means ± SEM of absorbance values at 450 nm for three technical replicates. (D–F) Total anti-CPS response of control sera from hyperimmunized mice. For titration of total anti-CPS antibodies, ELISA plates were not coated or coated with native CPS 7 (D), CPS 8 (E), or CPS 9 (F) and incubated with serial dilutions of sera, and antibodies were detected using specific HRP-conjugated anti-mouse total Ig [IgG + IgM]. Results are shown as means ± SEM of absorbance values at 450 nm for three technical replicates. Responses from wells that were not coated are shown by a grey circle, whereas the CPS-coated wells are shown by a colored diamond. (G–I) Total anti-*S. suis* response of sera from mice immunized with 25 µg of either CPS 7 (G), CPS 8 (H), or CPS 9 (I) native purified CPS adjuvanted with TiterMax Gold®. Mice (N = 10) were immunized on day 0 and boosted on day 21. Placebo mice (N = 10) were similarly injected with PBS adjuvanted with TiterMax Gold®. Sera were collected on day 42. ELISA plates were coated with whole bacteria of either *S. suis* serotype 7 strain 1750775 (G), serotype 8 strain 1719887 (H), or serotype 9 strain 1273590 (I) and incubated with sera samples diluted 1:100, 1:200, 1:400, or 1:800. Antibodies were detected using specific HRP-conjugated anti-mouse total Ig [IgG + IgM]. Results are shown as absorbance values at 450 nm for individual mice, with horizontal bars representing means ± SEM. Individuals from the placebo groups are shown by a grey circle, whereas those of the immunized groups are shown by a colored diamond.

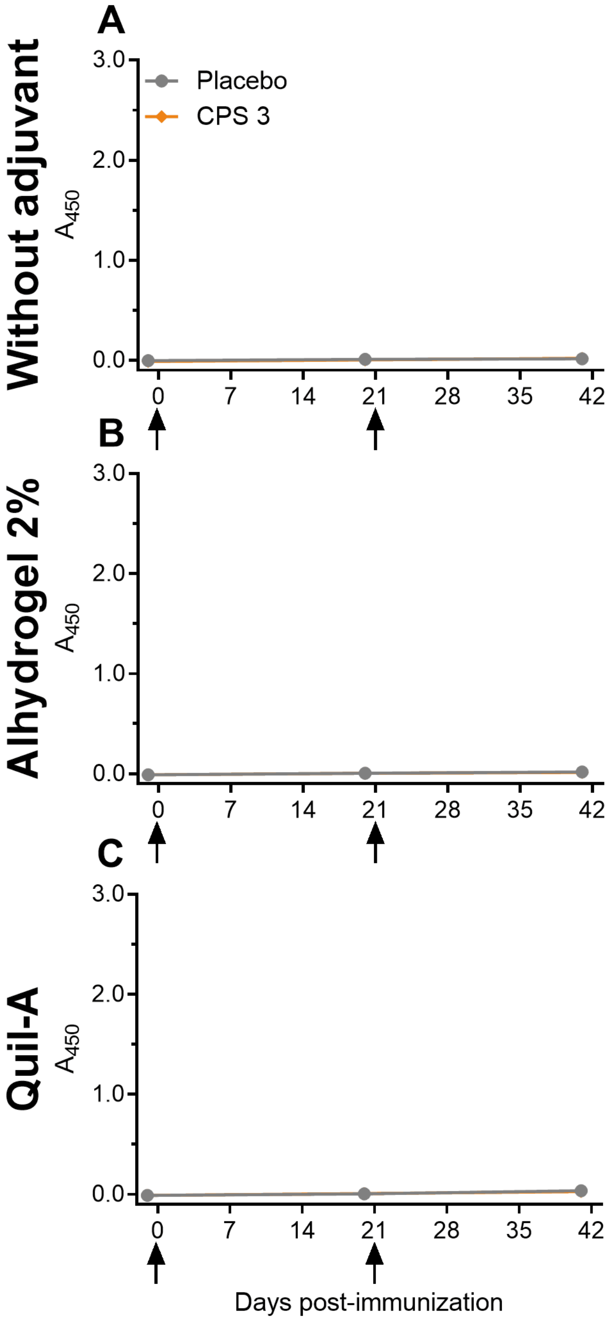

FIG S2 Kinetics of total antibody responses of mice immunized with 25 µg of native *S. suis* serotype 3 purified capsular polysaccharide (CPS 3) either non-adjuvanted or adjuvanted with Alhydrogel 2%® or Quil-A®. Mice (N = 10) were immunized on day 0 and boosted on day 21.

Placebo mice (*N* = 10) were similarly injected with PBS only or PBS adjuvanted with Alhydrogel 2%® or Quil-A®. ELISA plates were coated with native CPS 3 and incubated with blood samples diluted 1:100 to measure anti-CPS antibodies. Total Ig [IgG + IgM] antibody levels are shown as means ± SEM of absorbance values at 450 nm. Placebo groups are shown by a grey circle, whereas the CPS 3-immunized groups are shown by an orange diamond. The arrow at day 21 indicates the boost.

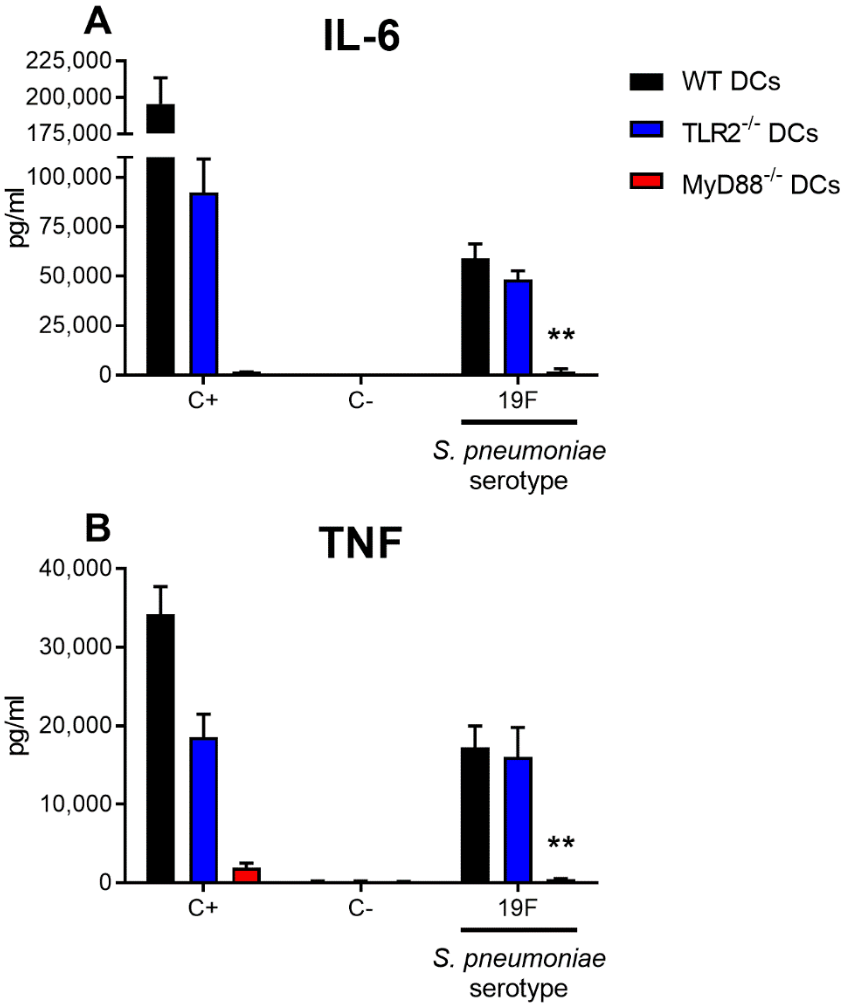

FIG S3 Role of TLR2 and of MyD88 in cytokine production by dendritic cells (DCs) in response to stimulation by *Streptococcus pneumoniae* CPS for 24 h. CPS of *S. pneumoniae* serotype 19F (at 200 µg/ml) was incubated with either wild-type (WT; black bars), TLR2$^{-/-}$ (blue bars) or MyD88$^{-/-}$ (red bars) DCs (10^6 cells/ml). After 24 h, supernatants were collected, and IL-6 (A) and TNF (B) levels were quantified by ELISA. Cells stimulated with medium alone and with LPS (1 µg/ml) served as negative (C-) and positive (C+) controls, respectively. Data are expressed as means ± SEM for at least three experiments. Statistically significant differences *versus* the WT are indicated as follows: **, $P \leq 0.01$.

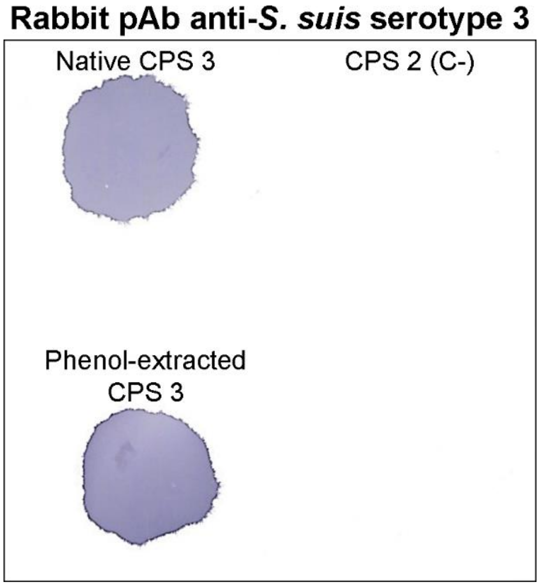

FIG S4 Dot-blot analysis with 10 μg of native and phenol-extracted capsular polysaccharides from *Streptococcus suis* serotype 3 (CPS 3) using rabbit anti-*S. suis* serotype 3 serum (pAb). Purified CPS from *S. suis* serotype 2 (CPS 2) was used as a negative control (C-).

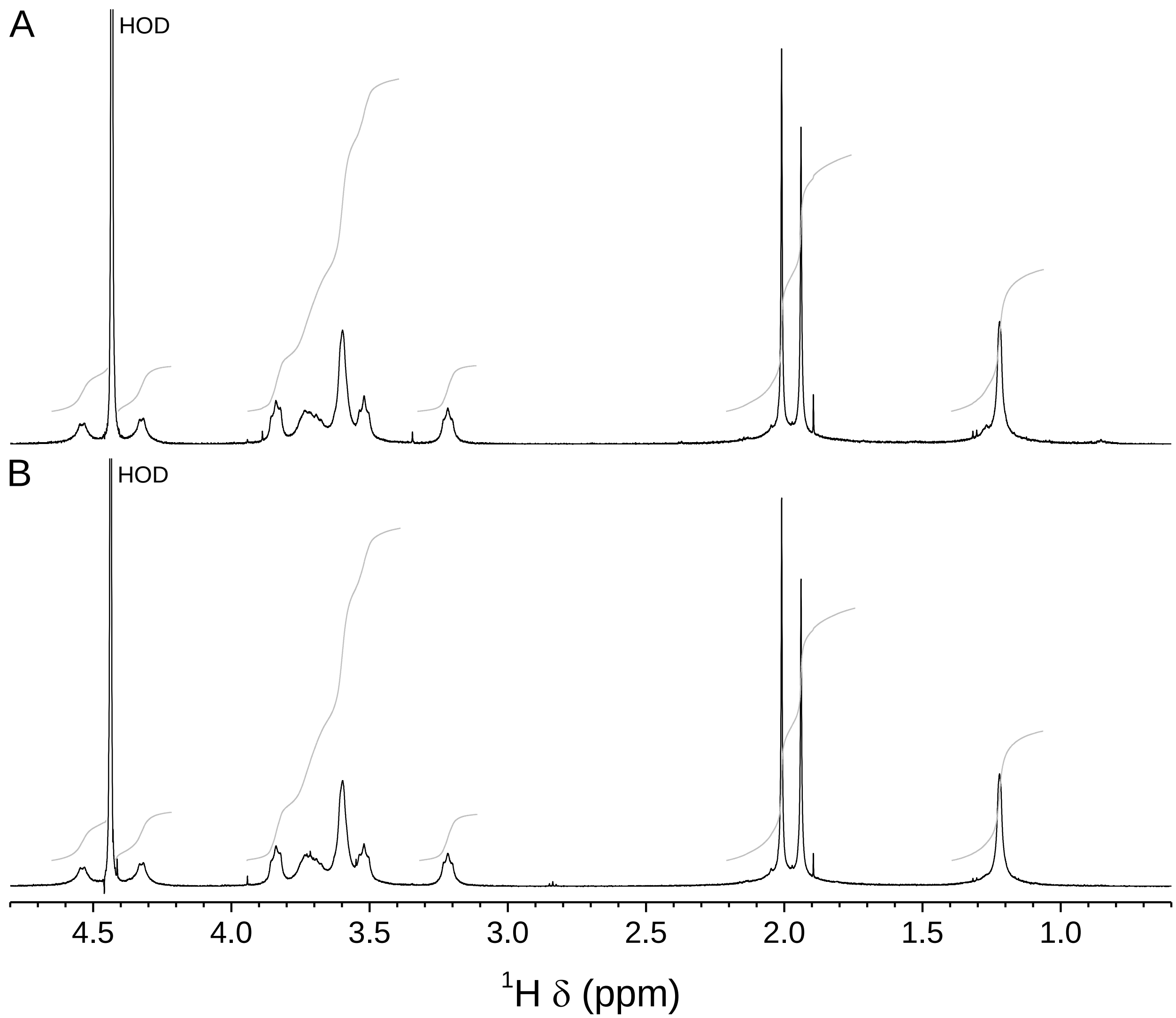

FIG S5. 500-MHz ^{1}H NMR spectra of *Streptococcus suis* serotype 3 native and phenol-extracted capsular polysaccharides (CPS 3) in D_2O at 60°C. (A) Native CPS 3. (B) Phenol-extracted CPS 3.
